# Investigation of Cytotoxicity Apoptotic and Inflammatory Responses of Biosynthesized Zinc Oxide Nanoparticles from *Ocimum sanctum* Linn in Human Skin Keratinocyte (Hacat) and Human Lung Epithelial (A549) Cells

**DOI:** 10.1155/2020/1835475

**Published:** 2020-08-12

**Authors:** Bader Almutairi, Gadah Albahser, Rafa Almeer, Nouf M. Alyami, Hanouf Almukhlafi, Khadijah N. Yaseen, Saad Alkahtani, Saud Alarifi

**Affiliations:** Department of Zoology, College of Science, King Saud University, Riyadh, Saudi Arabia

## Abstract

Pristine and engineered metal nanoparticles are widely applied in various fields of industry, and as consequences, they are useful as well as harmful to human health and environment. This study aimed at synthesizing the green zinc oxide nanoparticles (ZnNPs) using the leaf extract of *Ocimum sanctum* Linn and assessing its toxicity on human skin epidermal (HaCaT) and human lung epithelial (A549) cells. The synthesized green ZnNPs (gZnNPs) were characterized by using dynamic light scattering (DLS) and a high-resolution transmission electron microscope. The average size of gZnNPs obtained was 62 nm with a spherical shape. The effects of gZnNPs on the viability of HaCaT and A549 cells were investigated using tetrazolium salt (MTT) for 24 h. We have seen more reduction of cell viability of A549 cells in comparison to HaCaT cells. The induction of intracellular reactive oxygen species (ROS) was measured using DCFDA assay and showed a slightly high intensity of green fluorescence in A549 than HaCaT cells. The different oxidative stress biomarkers such as ROS generation and lipid peroxide were increased, and GSH was decreased in a dose-dependent manner. The apoptotic and necrotic effect of gZnNPs in both cells was carried out using Annexin-V-FITC and propidium iodide staining. More apoptotic and necrotic cells were found at a higher concentration of gZnNPs exposure. Also, we determined the effect of gZnNPs at the molecular level by evaluating the apoptotic and inflammatory markers, in which gZnNPs downregulated Bcl2 and upregulated Bax, caspase-3, and TNF-*α* in HaCaT and A549 cells. Ultimately, gZnNPs exerted toxicity and apoptosis in HaCaT and A549 cells.

## 1. Introduction

Engineered nanoparticles are an inventive class of materials. Due to this reason, development of nanotechnology is used in different interdisciplinary areas, e.g., medical purposes and industry. In this field, the improvement of nanotechnology headed to the productions of nanoparticles (NPs), as contrast agents or in targeted treatments. Recently, due to their more applications in clinical purposes, the NPs are intensively investigated, and more development was done on new techniques to biological synthesize eco-friendly nanomaterials and on assessing their biological effects on living organisms [1]. The manufacturing techniques of eco-friendly NPs are a challenge that made nanotechnology one of the most studied and well-financed domains of the last decades. Metal or metal oxide NPs are progressively used in dermatology and cosmetology. For example, ZnO and TiO2 NPs have been extensively used to sunscreens since the 1980s to confer better ultraviolet (UV) protection than traditional inorganic sunscreens [2]. Natural antioxidants are in high demand for application as nutraceuticals, biopharmaceuticals, as well as a food additive [3]. *Ocimum sanctum* Linn is commonly known as Tulsi and is applied for the curing of various health indications. However, *Ocimum sanctum* has medicinal properties and used as herbal tea. Chandrasekaran et al. [4] have reported its signs of toxicity for 14 days in Wistar rats. In the current investigation, the leaf extracts of *Ocimum sanctum* were used to biologically synthesize of ZnONPs and mediate its toxicity involved various mechanisms, in particular the production of excess ROS. As it is well known, mitochondrial dysfunction is the major source of ROS overload [5]. Oxidative stress, apoptotic, and inflammatory response are key molecular mechanisms to induce toxic effects of exogenous substances [6, 7]. Oxidative stress mainly results from the generation of intracellular ROS. Excessive ROS causes the imbalance of oxidation and antioxidant system in the body, which may induce lipid peroxidation and change various enzymatic activities [8]. Malondialdehyde is one of the most important products of lipid peroxidation, which can be measured to reflect cell oxidative damage. However, antioxidant enzyme superoxide dismutase (SOD) that catalyzes SOD could catalyze superoxide anion radicals to oxygen and hydrogen peroxide, and then, catalase (CAT) catalyzes hydrogen peroxide to oxygen and water, helps to scavenge ROS and free radicals, and prevents the cells from an injury effectively. Studies showed that oxidative stress is often associated with the germination of inflammation. ROS generated by ZnNPs not only induced oxidative damage but also increased the synthesis of proinflammatory cytokines in human epithelial cells. Few studies have confirmed the harmful effects of gZnNPs on human skin epidermal cell lines. So, in this study, we will investigate the toxic effects of gZnNPs on HaCaT and A549 cells.

## 2. Materials and Methods

### 2.1. Chemical and Reagents

Green zinc oxide nanoparticle (gZnNPs) was prepared by using leaves extracts of *Ocimum sanctum* Linn plant. MTT [3-(4, 5-dimethylthiazol-2-yl)-2, 5-diphenyltetrazolium bromide], DCFH-DA, dimethyl sulfoxide, Annexin-V-FITC, and propidium iodide were purchased from Sigma-Aldrich (St. Louis, Missouri, United States). Dulbecco's modified Eagle's medium (DMEM), fetal bovine serum (FBS), and antibiotics were purchased from Gibco, USA.

### 2.2. Preparation of Leaves of *Ocimum sanctum* Extracts

The fresh leaves of *Ocimum sanctum* Linn were collected from the local area and washed with running tap water. Leaves were cut in small pieces, ground, and filtered using mesh (22 *μ*m) and stored in the refrigerator for further use.

### 2.3. Green Synthesis of ZnNPs and Physical Characterization

The leaves extracts (50 g/l) were mixed to 5 g/l of zinc acetate in a conical flask and very well shacked. After 30 min, the reaction mixture was heated overnight at 60°C, and the ZnO NPs powder is obtained.

A measurement of synthesized gZnNPs was done by using a high-resolution transmission electron microscope (JEOL Inc., Tokyo, Japan) at an accelerating voltage 120 kV. The size of gZnNPs in water and culture medium were determined by DLS (Nano-Zeta Sizer-HT, Malvern, UK) as described by Alarifi et al. [9]. We have used 35 *μ*g/ml for DLS measurement because this is the maximum treatment concentration used to evaluate cell viability.

### 2.4. Establishment of Cell Lines and Exposure of gZnNPs

HaCaT and A549 cells were brought from American Type Culture Collection (ATCC), USA. These cells were cultured in DMEM with 10% FBS and 10000 U/ml antibiotics at 5% CO_2_ incubator at 37°C. The cells at 80% confluence were subculture into 96 well plates, 6 well plates, and 25 cm^2^ flasks according to designed experiments.

HaCaT and A549 cells were subcultured for one day before treatment to gZnNPs. The standard solution of gZnNPs was prepared in Milli-Q water at 1 *μ*g/*μ*l and diluted according to the experimental dosage (0-35 *μ*g/ml). Control cells were not exposed to NPs and considered as controls with each experiment.

### 2.5. Cell Viability Detection

The cell survival rates were measured by methylthiazol tetrazolium (MTT) assay after 24 hours of exposure. Briefly, HaCaT and A549 cells in good growth conditions were collected and seeded into 96-well plates at 5 × 10^3^ cells/well. Four multiple wells were prepared for each concertation. After adhesion, the cells were exposed to various concentrations of gZnNPs (0, 2, 6, 12, 25, and 35 *μ*g/ml). Meanwhile, an equal volume of the medium was used as a blank control, and cell suspension alone as controls. After exposure for 24 h, 10 *μ*l MTT (5 mg/ml) was added into each well and cultured for another 4 hours. Then, 100 *μ*l SDS-isopropanol-HCL (triple solution) was added to each well and placed overnight at 37°C. The absorbance at 570 nm was detected by a microplate reader (BioTek, USA) to calculate cell viability.

### 2.6. Estimation of Intracellular ROS Production

Formation of ROS in HaCaT and A549 cell lines due to gZnNPs (0, 6, 12, 25, and 35 *μ*g/ml) treatment for 24 h was determined according to the method [10]. Briefly, 1 × 10^4^ HaCaT and A549 cells were grown in 96-well black culture plates and incubated in a CO_2_ incubator at 37°C for 24 h for attachment. Different concentrations of gZnNPs were added to the culture plates of HaCaT and A549 cells for 24 h. After exposure, the culture plates were washed with PBS, and 10 *μ*M DCFH-DA was mixed per well at 37°C for 60 minutes. After incubation, the plate was washed and the fluorescence intensity was read at 485 nm excitation and 520 nm emissions using the microplate reader (Synergy-H1; BioTek). Data were represented as obtained fluorescence intensity of exposed and control wells.

A parallel set of experiments was carried out in a 6-well culture plate, and DCF fluorescence images of cells were captured by using a fluorescence microscope (Olympus CKX 41; Olympus: Center Valley, Pennsylvania, USA).

### 2.7. Detection of MDA Content and GSH Activities

After treatment with gZnNPs (0, 6, 12, 25, and 35 *μ*g/ml) for 24 h, the cells were collected by 0.25% trypsin and split by ultrasonic crushing. After that, the cell samples were used for the next detection. The content of GSH and the MDA was measured according to the instruction of Cayman Chemical kits.

### 2.8. Caspase-3 Activity

Caspase-3 enzymes play a significant role in cell death. Caspase-3 enzymes were determined in gZnNPs (0, 6, 25, and 35 *μ*g/ml) exposed and control cells by using the BioVision colorimetric assay kits.

### 2.9. Analysis of Apoptosis Using FCM

The apoptotic and necrotic HaCaT and A549 cells due to gZnNP (0, 6, 25, and 35 *μ*g/ml) exposure were detected using Annexin-V-FITC/PI staining through flow cytometer (Becton-Dickinson Immunocytometry Systems, Sunnyvale, CA, USA). The cells were collected by trypsin enzyme and washed with PBS and resuspended in binding buffer. Annexin-V-FITC (5 *μ*l) and PI (5 *μ*l) were mixed to cell suspension (500 *μ*l) and put in the dark for 30 minutes at room temperature. Fluorescence emitted by Annexin-V-FITC and DNA-bound propidium iodide in each event were detected as red fluorescence, respectively. Results were analyzed by the FACS Diva 6.1.2 software.

### 2.10. RNA Isolation and RT qPCR

The total RNA was extracted from gZnNP- (0, 6, 25, and 35 *μ*g/ml) treated HaCaT and A549 cells using the RNAiso Plus reagent (Takara, China) after treatment for 24 h, and the concentration was measured at the absorbance of 260 nm by NanoDrop 2000C spectrophotometer (Thermo Scientific, Waltham, Massachusetts). Then, the total RNA was reverse-transcribed into single-strand cDNA using the PrimeScript RT reagent kit (Takara, China). The gene expression levels were quantified using a SYBR Premix Ex Tap II Kit in the IQ5 Real-Time PCR Detection System (Bio-Rad, USA) under the conditions of heating at 95°C for 30 s, 40 cycles of denaturing at 95°C for 5 s, annealing at 55-60°C for 30 s, and extension at 72°C for 30 s. The primer sequences are shown in Table 1, *β*-actin was used as the internal reference, and the relative mRNA expression was calculated by using the 2^-*ΔΔ*Ct^ method.

### 2.11. Western Blot Assay

HaCaT and A549 cells were treated with gZnNPs (0, 25, and 35 *μ*g/ml) for 24 h, and after exposure, the cell lysate was prepared in RIPA buffer (ab156034). The cell lysate was centrifuged at 13000 rpm, 4°C for 30 min, and the supernatant was used for protein expressions. The concentration of protein was evaluated by the Bradford method [11]. Protein (20 *μ*g) was migrated on the gel and transferred to a PVDF membrane (Bio-Rad, Laboratories Inc., Berkeley, CA, USA). The PVDF membrane was incubated with different mouse monoclonal antibody against *β*-actin (1 : 12000 dilutions, Abcam, Cambridge, UK), Bcl2 (1 : 500 dilutions, Santa Cruz), Bax (1 : 1000 dilutions, Antibodies-online), Caspase-3 (1 : 500 dilutions, Cayman), and TNF-*α* (1 : 500 dilutions, Santa Cruz) for 24 h at 4°C.

Secondary antibody HRP-conjugated goat anti-mouse IgG (H + L) antibody (1 : 2000 dilutions Bio-Rad) was used. Immunoreactive bands were detected using an EZ west Lumi plus (ATTO Corporation, Tokyo, Japan), which is a chemiluminescent substrate to detect HRP on the western blotting membrane. The luminescence intensity (optical density) of the target protein bands was quantified using Lumino Graph 2 (ATTO Corporation). All protein expression levels were normalized to the levels of *β*-actin protein expression in each band.

### 2.12. Statistical Analysis

The data were analyzed by the SPSS 24.0 software (IBM) and expressed as mean ± standard deviation (SD). Differences between the groups were determined by a one-way ANOVA test with the least-significant difference test. Values of *p* < 0.05 were considered statistically significant.

## 3. Results

### 3.1. Characterization of gZnNPs

**Figure 1:**
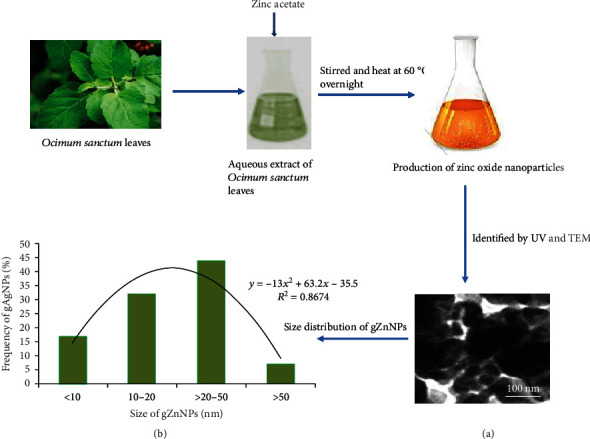
Schematic diagram of green synthesis of zinc oxide nanoparticles (gZnNPs) using zinc acetate salt and *Ocimum sanctum* leaf extract. (a) TEM image of gZnNPs. (b) Size distribution of gZnNPs (%).

Biosynthesized gZnNPs were characterized using HR-TEM and DLS (Figure 1). The TEM image of gZnNPs was presented in Figure 1(a). The average diameter of gZnNPs with spherical shape was 58.5 ± 2.5 nm (Figure 1(b)). The size of gZnNPs in dH_2_O and culture media was 140 ± 1.0 nm and 155 ± 2 nm. The zeta potential of NPs in dH_2_O and culture media were ~10.2 ± 0.8 mV and ~9.0 ± 1.6 mV (Table 2).

### 3.2. Cytotoxicity

**Figure 2:**
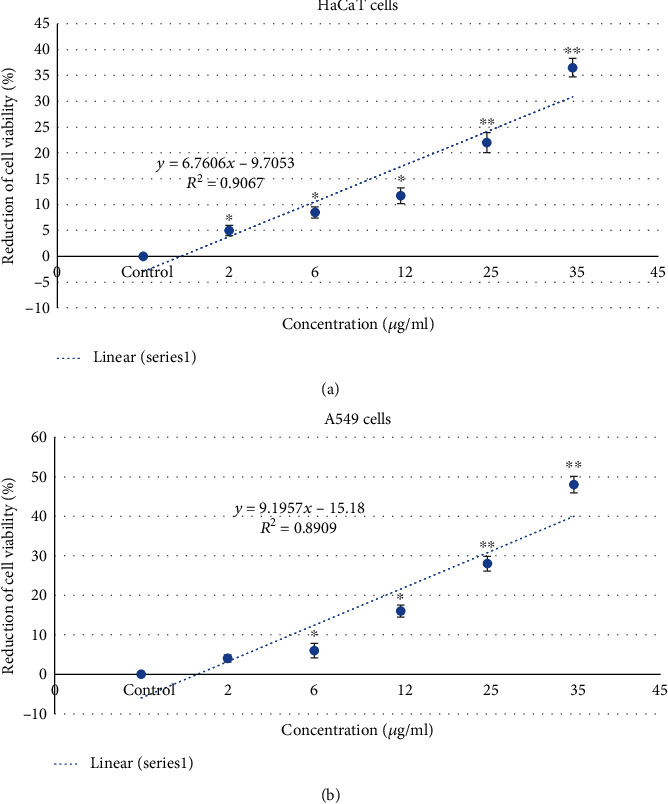
Cytotoxicity of gZnNPs on HaCaT and A549 cells for 24 h, as measured by MTT assay. Each value represents the mean ± SE of three experiments. *n* = 3, ∗*p* < 0.05, ∗∗*p* < 0.01 vs. control.

Cell viability of HaCaT and A549 cells were determined by the MTT test, and the results are presented in Figures 2(a) and 2(b). We have observed the reduction of cell viability in both cells in concentration in a dependent manner, and the A549 cell viability is more reduced than HaCaT cells Figures 2(a) and 2(b). We have found the reduction of cell viability as 0.02, 5%, 8.5%, 11.72%, 22%, and 36.5% in HaCaT cells (Figure 2(a)) and 0.03, 4%, 6%, 16%, 28%, and 48% in A549 cells (Figure 2(b)) at 0, 2, 6, 12, 25, and 35 *μ*g/ml gZnNPs, respectively.

### 3.3. Oxidative Stress

**Figure 3:**
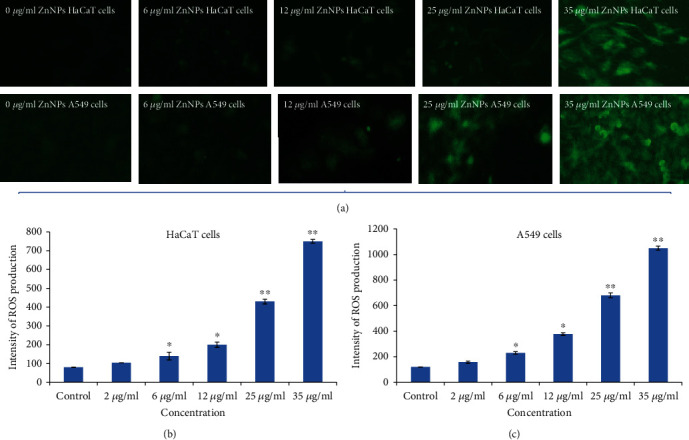
(a) Photomicrograph of green fluorescence (DCF intensity) in HaCaT and A549 cells after exposure to gZnNPs for 24 h. (b) Intracellular intensity of ROS production in HaCaT cells (c). Intracellular intensity of ROS production in A549 cells. Each value represents the mean ± SE of three experiments. ∗*p* < 0.05 and ∗∗*p* < 0.01 vs. control.

ROS production in HaCaT and A549 cells due to gZnNPs exposure was determined, and its generation depends on the concentration of NPs. The generation of ROS was measured by using DCFDA, and it was oxidized in cells and formed DCF fluorescent compound. High intensity of green fluorescence demonstrates more generation of ROS in cells. We have seen more generation of ROS in A549 cells than HaCaT cells (Figures 3(a)–3(c)). The generation of ROS was as DCF intensity was more in A549 cells (Figure 3(b)) than HaCaT cells (Figure 3(c)).

**Figure 4:**
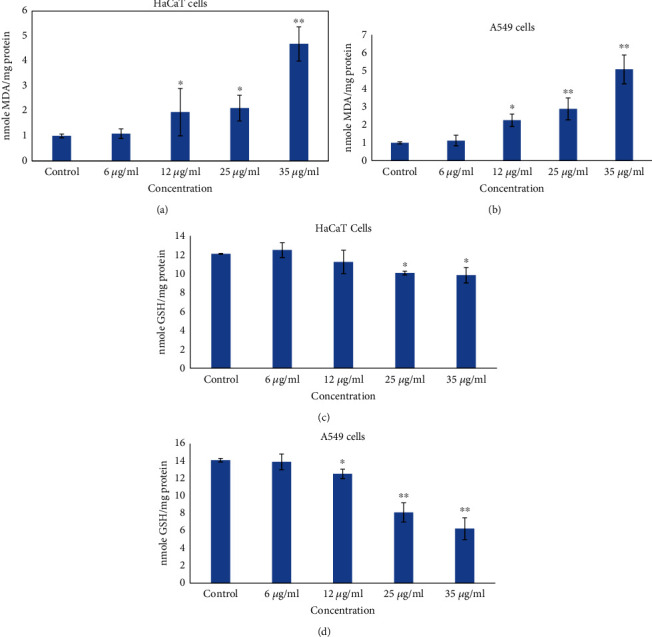
Oxidative stress biomarkers after exposure to gZnNPs on HaCaT and A549 cells for 24 h. (a) LPO in HaCaT. (**b**) LPO in A549 cells (c). GSH in HaCaT cells. (d) GSH in A549 cells. Each value represents the mean ± SE of three experiments. ∗*p* < 0.05 and ∗∗*p* < 0.01 vs. control.

Meanwhile, LPO and GSH were determined and compared with the control group, the activities of LPO was significantly increased in HaCaT (Figure 4(a)) and A549 cells (Figure 4(b)), and the GSH level was decreased in HaCaT (Figure 4(c)) and A549 cells (Figure 4(d)) at 35 *μ*g/ml after exposing ZnNPs.

### 3.4. Detection of Apoptotic

**Figure 5:**
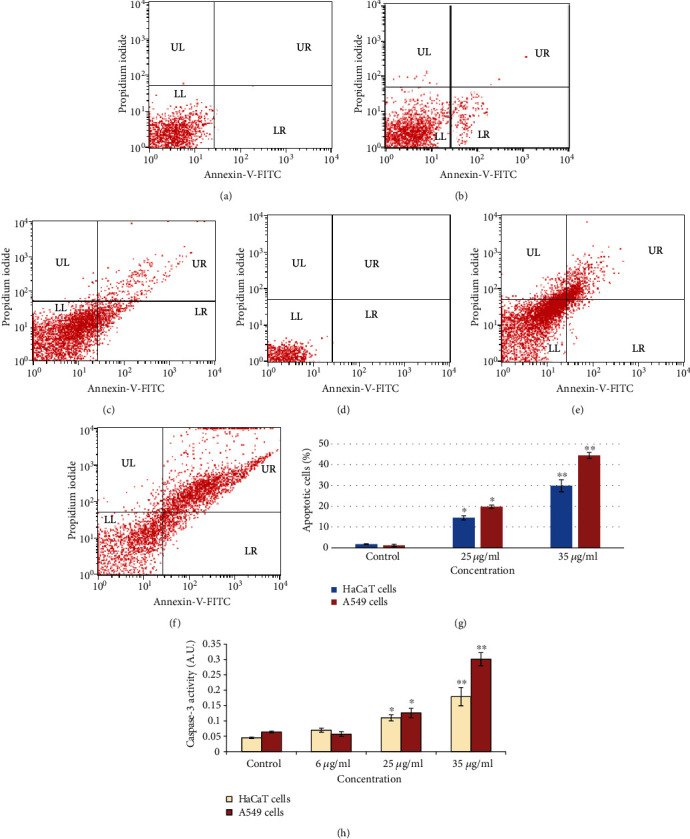
Scatter diagram after treatments of gZnNPs to HaCaT and A549 cells for 24 h. (a) HaCaT cells at 0 *μ*g/ml. (b) HaCaT cells at 25 *μ*g/ml. (c) HaCaT cells at 35 *μ*g/ml. (d) A549 cells at 0 *μ*g/ml. (e) A549 cells at 25 *μ*g/ml. (f) A549 cells at 35 *μ*g/ml. (g) Percentage of early and late apoptotic cells after treatment of ZnNPs (0, 25, and 35 *μ*g/ml) for 24 h. (h) Caspase-3 activity after treatment of ZnNPs (0, 6, 25, and 35 *μ*g/ml) for 24 h. Data represents the mean ± SE of three experiments. ∗*p* < 0.05 and ∗∗*p* < 0.01 vs. control.

The activity of caspase-3 was calculated in HaCaT and A549 cells. Activities of caspase-3 were increased according to concentration dependence, and its level was more in A549 cell than HaCaT cells (Figure 5(h)). We have confirmed the apoptotic effect of NPs on both cells using Annexin-V-FITC and PI staining, and the cell scatter diagram of cell distribution is shown in Figures 5(a)–5(g). Analysis of the percentage of early and late apoptotic cells was determined by (FACS) as shown in Figure 5(g). The early and late apoptosis rates were 44.65 in A549 cells and 29.84% in HaCaT cells at gZnNPs (35 *μ*g/ml) exposure, respectively, compared with the control group (Figure 5(g)).

### 3.5. mRNA Expressions in HaCaT and A549 Cells

**Figure 6:**
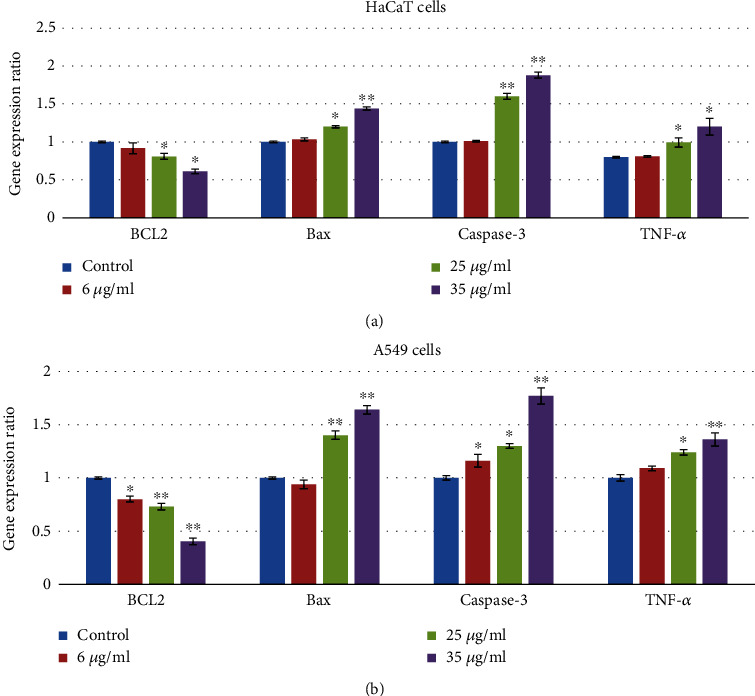
ZnNPs induce apoptosis and inflammatory response of HaCaT and A549 cells. Western blot analysis was carried out to assess apoptosis and inflammatory response-related protein expression in HaCaT and A549 cells. *β*-Actin was used as an internal reference. ZnNPs significantly increased the expression of Bax, caspase-3, and TNF-*α* and decreased the expression of Bcl-2 at a concentration 35 *μ*g/ml (a, b). Data represents the mean ± SE of three experiments. ∗*p* < 0.05 and ∗∗*p* < 0.01 vs. control.

**Figure 7:**
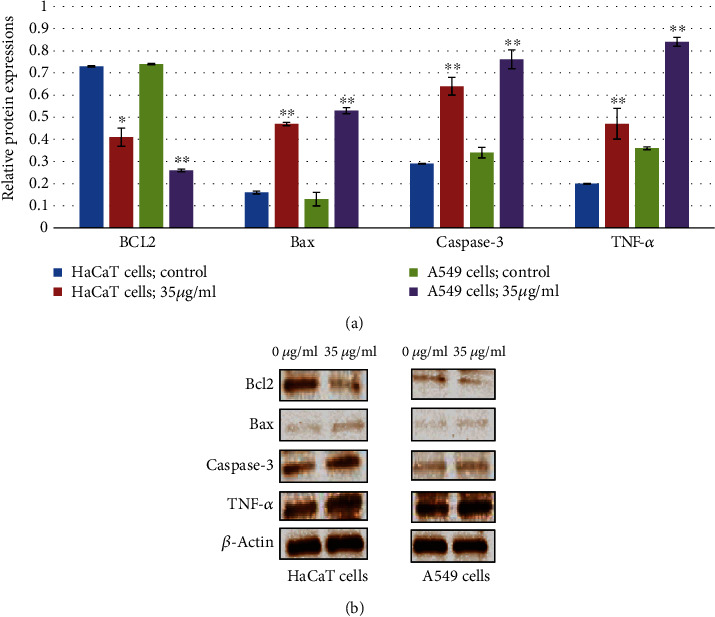
ZnNPs induce apoptosis and inflammatory response of HaCaT and A549 cells. mRNA expression analysis was carried out to assess apoptosis and inflammatory response-related gene expression in HaCaT cells (a) and A549 cells (b). *β*-Actin was used as an internal reference. ZnNPs significantly increased the gene expression of Bax, caspase-3, and TNF-*α* and decreased the expression of Bcl-2 in a concentration-dependent manner (a, b). Data represents the mean ± SE of three experiments. ∗*p* < 0.05 and ∗∗*p* < 0.01 vs. control.

The mRNA expressions of Bax, Bcl2, caspase-3, and TNF-*α* were identified to confirm apoptotic and inflammatory effects of gZnNPs (Figure 6). Compared with the control group, the mRNA expressions of Bax, caspase-3, and TNF-*α* were increased while expression of Bcl2 was decreased at 35 *μ*g/ml gZnNPs group in HaCaT (Figure 7(a)) and A549 cells (Figure 7(b)).

### 3.6. Western Blot Analysis

The expression of apoptotic and inflammatory proteins in HaCaT and A549 cells after gZnNPs exposure was determined using western blot analysis. In HaCaT cells, gZnNPs reduced in Bcl2 protein expression (Figures 6(a) and 6(b)) and increased expression of Bax, caspase-3, TNF-*α*, and IL-6 as the compared control group (Figures 6(a) and 6(b)). Also, similar effects of gZnNPs on the expression of apoptotic and inflammatory proteins were observed in A549 cells (Figures 6(a) and 6(b)). The results indicated higher expressions of apoptotic and inflammatory protein in A549 cells than HaCaT cells (Figures 6(a) and 6(b)).

## 4. Discussion

Nanosize particles and dust are key risk factors for respiratory diseases and distribute in ambient air at significant levels. Nanotechnology is an inventive area of science with more application of new materials. In this study, ZnNPs was synthesized using the extract of *Ocimum* leaves and used to explore the potential influence of exposure to ZnNPs on the skin and pulmonary inflammation in HaCaT and A549 cells, respectively. TEM analysis explored that the average size of synthesized ZnONPs was 58.5 ± 2.5 nm with a face-centered cubic structure with a spherical shape (Figure 1). Moosa et al. [12] and Ahmed et al. [13] reported that the size of synthesized nanoparticles depends upon the plant extract volume. No cytotoxicity was observed at a lower concentration, while exerting clear cytotoxic effects at higher concentration of gZnNPs (at 35 *μ*g/ml) for 24 h in HaCaT and A549 cells. These results suggested the cytotoxic effects of gZnNPs to some extent. Alarifi et al. [14] reported zinc oxide nanoparticle (at 20 *μ*g/ml) induced cytotoxicity, oxidative stress, and apoptosis in malignant human skin melanoma cell line. Also, this study showed that gZnNPs inhibited cell viability more in A549 cells than HaCaT cells. Furthermore, gZnNPs showed a low reduction of cell viability in HaCaT and A549 cells than the reduction of cell viability in A375 cells due to ZnONP exposure. This might occur in the presence of an extract of *Ocimum sanctum*. Gautam and Goel [15] reported that 50% ethanol extract of *Ocimum sanctum* leaves was nontoxic after its acute and subacute oral administrations mice. Nowadays, biosynthesized metallic nanoparticles using plant extract have received more attention due to simple and viable alternative against chemical and physical methods with their potential applications in nanomedicine. Zinc oxide nanoparticles were synthesized using *Laurus nobilis* L leaf aqueous extract and two different zinc salts [16] and showed antibacterial activity [17]. In this experiment, we have investigated the generation of intracellular reactive oxygen species, and its levels increase in a dose-dependent manner; subsequently, ROS induction was more in A549 cell than HaCaT cells. Conformably, the current results showed significant generations of ROS and MDA by ZnNPs in A549 and HaCaT cells. Nanoparticles induced free radicals which may damage the cells through oxidative stress mechanism. The generation of intracellular ROS deteriorates the lipid membrane of target cells by the formation of malondialdehyde molecule, and this is known as lipid peroxidation [18]. In this study, LPO activity was observed more in A549 than in HaCaT cells. ROS has been linked in different mechanisms, viz. damaging of nuclear components (DNA and RNA), protein, interference of cellular signaling pathways, and alteration in gene expression, and ultimately, the mechanism of cell growth was stopped [18]. Interestingly, among these two cell types, A549 cells were more sensitive than HaCaT cells. Liu et al. [19] found that particulate matters increased intracellular ROS level and induced oxidative stress via the activation of IL-6/AKT/STAT3/NF-*κ*B signaling pathway in lung epithelial cells. To confirm whether proliferation of cell was inhibited by the apoptotic response, we have determined apoptotic cells by using Annexin-V-FITC and PI staining after exposure to gZnNPs. The gZnNP-induced apoptosis was led by the activation of caspase-3, which is observed at 35 *μ*g/ml nanoparticle exposure by evaluation of caspase-3 activity by the enzyme-linked immunosorbent assay (ELISA) and immunoblotting. Activation of caspase-3 was accompanied by downregulation of bcl2 and upregulation of Bax proteins. All these events indicated the signs of apoptosis, which were observed more in A549 cells after exposure to NPs.

It is a more important finding that A549 cells are more susceptible to gZnNPs than HaCaT cells. In the future, we will do investigations about the mechanism of toxicity due to gZnNPs in in vivo experiments.

## Figures and Tables

**Table 1:** Primer sequences and theoretical amplification length.

Genes	Orientation primer	Sequences (5′ to 3′)
Bcl-2	Forward-	ATGTGTGTGGAGACCGTCAA
	Reverse-	GCCGTACAGTTCCACAAAGG
BAX	Forward-	ATGTTTTCTGACGGCAACTTC
	Reverse-	AGTCCAATGTCCCAGCCCAT
Caspase-3	Forward-	TGTTTGTGTGCTTCTGAGCC
	Reverse-	CACGCCATGTCATCATCAAC
TNF-*α*	Forward-	AAGGACACCATGAGCACTGAAAGC
	Reverse-	AGGAAGGAGAAGAGGCTGAGGAAC
*β*-Actin	Forward-	CCTGGCACCCAGCACAAT
	Reverse-	GGGCCGGACTCGTCGTCATAC

**Table 2:** Hydrodynamic size and zeta potential of gZnNPs in different dispersion medium. Data represent the mean ± SE of three independent experiments.

Nanopowder	Dispersion media	DLS mean ± SE (nm)	Zeta potential ± SE (mV)
gZnNPs	d-H_2_O	140 ± 1.0	~10.2 ± 0.8
	DMEM/supplemented media	155 ± 2	~9.0 ± 1.6

## Data Availability

All data generated or analyzed during this study are included in the article.
